# The influence of ego depletion on sprint start performance in athletes without track and field experience

**DOI:** 10.3389/fpsyg.2015.01207

**Published:** 2015-08-17

**Authors:** Chris Englert, Brittany N. Persaud, Raôul R. D. Oudejans, Alex Bertrams

**Affiliations:** ^1^Department of Educational Psychology, University of Bern, Bern, Switzerland; ^2^Department of Human Movement Sciences, MOVE Research Institute Amsterdam, VU University, Amsterdam, Netherlands

**Keywords:** ego depletion, reaction time, self-control, self-regulation, sprint

## Abstract

We tested the assumption that ego depletion would affect the sprint start in a sample of *N* = 38 athletes without track and field experience in an experiment by applying a mixed between- (depletion vs. non-depletion) within- (T1: before manipulation of ego depletion vs. T2: after manipulation of ego depletion) subjects design. We assumed that ego depletion would increase the possibility for a false start, as regulating the impulse to initiate the sprinting movement too soon before the starting signal requires self-control. In line with our assumption, we found a significant interaction as there was only a significant increase in the number of false starts from T1 to T2 for the depletion group while this was not the case for the non-depletion group. We conclude that ego depletion has a detrimental influence on the sprint start in athletes without track and field experience.

## Introduction

For elite level performance in the track and field 100 meter dash it is decisive to initiate the sprinting motion as quickly as possible following the starting signal (e.g., Santana, 2000; Gough, 2006; Pilianidis et al., 2012), as for instance Harland and Steele (1997) have shown that the sprint start accounts for roughly 5% of the total race time. At the same time, potential false starts need to be averted as it could mean getting disqualified from the competition (e.g., Ditroilo and Kilding, 2004). Previous research has demonstrated that the quality of the sprint start is essential for peak performance (e.g., Brown and Vescovi, 2012). International Association of Athletics Federations (2013) established a false start criterion to make sure that athletes do not “guess” when to start and thus initiate their sprinting motions too early. This false start criterion is violated if athletes initiate their sprinting motion less than 100 ms after the starting signal has been given.

When being in the starting block an athlete wants to gain an edge by delivering a perfect start (Collet, 1999), while at the same avoiding a false start as it could mean immediate disqualification. We assume that inhibiting this starting impulse and also initiating the movement are both acts that require self-control, an assumption we will explain in more detail below. Self-control means the ability to volitionally override predominant response tendencies in order to achieve a specific goal (e.g., Baumeister et al., 1998). In their strength model of self-control Baumeister et al. (1998) postulate that self-control acts are based on one global, metaphorical resource, the so-called self-control strength (e.g., Baumeister et al., 1998; Muraven and Baumeister, 2000). Self-control strength is not domain-specific as all self-control acts (e.g., emotion regulation, persistence) are energized by the same resource (e.g., Muraven and Baumeister, 2000). For instance a primary cognitive task requiring self-control can negatively influence performance of tasks in different domains (e.g., emotion regulation; Englert and Bertrams, 2013). The capacity of this resource is limited, however, and can become temporarily depleted after previously exerting self-control, which is a temporary state labeled ego depletion (e.g., Baumeister et al., 1994). There is a carry-over effect on subsequent self-control performance, as individuals do not perform up to their capabilities in a state of ego depletion (for an overview, see the meta-analysis by Hagger et al., 2010). In the domain of sport psychology previous research has demonstrated that individuals in a state of ego depletion are less persistent in straining physical exercises (e.g., Martin Ginis and Bray, 2010), are more likely to choke under pressure (e.g., Englert and Bertrams, 2012; Englert et al., 2015), and even display lower performance in well-elaborated exercises (e.g., pushups; Dorris et al., 2012).

The sprint start also requires self-control strength as there are two competing impulses that need to be volitionally regulated: On the one hand, the impulse to start too soon needs to be regulated. As previously mentioned, while being in the starting block an athlete wants to gain an edge by initiating the movement immediately after the starting signal. Therefore the predominant response tendency would be to initiate the movement at the risk of displaying a false start and self-control strength would be necessary to volitionally downregulate this behavioral tendency in order to avoid false starts. This is in line with studies in which participants in a state of ego depletion were less adept in controlling their motor impulses (e.g., Finkel et al., 2006; McEwan et al., 2013; Graham et al., 2014). On the other hand, reacting as quickly as possible after having received a signal is an act that is also based on self-control, especially if participants are well aware of the negative consequences of starting too early. In that case, the predominant response tendency would be to rather wait to initiate a movement in order to minimize the possibility of a false start. Self-control would then be necessary to volitionally override this behavioral tendency as quickly as possible. Indeed, several studies have demonstrated that reaction times can be impaired under ego depletion, as for instance ego depleted individuals display longer response latencies in the Stroop task (Stroop, 1935), which is also a task in which a predominant tendency (i.e., to name the color word) needs to be substituted by a different response (i.e., to name the font color instead; e.g., Richeson and Shelton, 2003).

In a recent study, Englert and Bertrams (2014) found that the reaction times in sprints depend on self-control strength. In their study athletes with experience in the track and field 100 meter dash, displayed slower reaction times in a state of ego depletion as compared to a state with temporarily available self-control strength. Interestingly, there was no effect of ego depletion on the number of false starts. The authors reasoned that experienced sprinters are well aware of the drastic negative consequences of false starts (i.e., disqualification), which led to a dominance of the tendency to rather “wait” a little longer in the starting block, than to risk the possibility of a disqualification by initiating the motion too early. Therefore, in experienced athletes self-control strength would be necessary to override this predominant waiting tendency which is why athletes in a state of ego depletion took longer to initiate their movements. According to Englert and Bertrams (2014) the level of expertise seemed to be an important factor in their study. Indeed, false starts are less likely in more experienced sprinters (Majumdar and Robergs, 2011; Pilianidis et al., 2012).

In the current study, we again tested the effect of ego-depletion on sprint performance but now of athletes without specific track and field sprinting experience, but with general sprinting experience, namely experienced female soccer players. We wondered whether an effect of ego depletion on false starts would emerge in this specific sample. It could be argued that in athletes without sprinting experience the response to suppress and avoid false starts may be less developed than in experienced track and field sprinters. Athletes without track and field experience may be less aware of the negative consequences of a false start. This would imply that for athletes without track and field experience the dominant response would always be to start as quickly as possible without the competing response to not initiate too early. This would mean that in a state of ego depletion they would have less self-control to suppress the strong movement response immediately before the starting signal, which in turn would increase the likelihood of false starts. The theoretical implication of such a finding would be that level of experience moderates how the detrimental effect of ego depletion on the sprint start occurs—as slowing down the starting speed in experienced athletes (Englert and Bertrams, 2014) or as instigating false starts in inexperienced athletes (the present study).

In a mixed between- (depletion vs. non-depletion) within- (T1 vs. T2) subjects design female soccer players performed a series of three 20-m sprints at baseline (T1) and following an ego depletion manipulation (T2). We measured the average reaction times (in ms) and the number of false starts at both times of measurement. As mentioned, we expected the number of false starts to increase in a state of ego-depletion.

## Materials and Methods

### Participants

Our sample consisted of 38 Collegiate level female soccer players who voluntarily participated in the experiment (*M*_age_ = 20.58, SD_Age_ = 2.10; years of soccer experience: *M* = 14.13, SD = 3.88). The results of a G*Power analysis demonstrated that our sample size was sufficient to detect at least a medium effect in the current study (Faul et al., 2007; parameters: *f* = 0.25, α = 0.05, 1-β = 0.80, *r*_repeated measures_ = 0.50, ε = 1). A previous health screening did not reveal any physical impairments in any of the participants and no participant had performed any straining physical activity beforehand. Participants were randomly assigned to a depletion group (*n* = 19 depletion) or a non-depletion group (*n* = 19). We obtained written informed consent from each participant before the experiment and the study was approved by the local ethics committee and carried out in accordance with the Helsinki declaration of 1974.

### Materials and Procedure

The study was conducted by the same experimenter on the training facilities of the respective soccer club. Participants first reported demographic information (i.e., age, sex, years of soccer experience) and completed an individual warm up phase of approximately 5–10 min.

Then, participants were familiarized with the sprint protocol. Subjects were directed to stand on a touch-and-release pad from which the reaction times were recorded (e.g., Haugen et al., 2012). The auditory stimuli was delivered via a Brower Timing System (Brower, Salt Lake City, UT, USA) synched with a start timer. The Brower Timing System has proven to be a reliable measure of reaction times (e.g., Hetzler et al., 2008). The sprint protocol began when the Brower Timing System provided an acoustic starting signal. In line with the guidelines of the International Association of Athletics Federations (2013) the timeframe “set” and the start signal was 1–3 s and was randomly chosen for each sprint to minimize the likelihood of guessing when to start. The reaction times were measured from the acoustic starting signal to the time when the pressure from the front foot against the touch-and-release pad was removed. The participants first completed three 20-m practice trials at 50% maximum speed with the addition of an auditory stimulus, to allow the individual to become familiar with the system. This trial protocol was completed at a submaximal level so that fatigue would not influence the participant’s subsequent sprint performance during the experimental trials. After a short recovery, as baseline measure (T1) participants then performed three maximum speed 20 m sprints and were instructed, upon hearing the acoustic signal, to sprint as quickly as possible, from the touch-and-release pad. Following each trial, participant’s passively recovered for 90 s (Frost et al., 2008). Reaction times and false starts were derived for each experimental trial, with reaction times below 100 ms also counted as false starts.

During the adjacent recovery phase the participants completed several self-report measures. We measured participants’ level of trait self-control strength by administering the brief Self-Control Scale (Tangney et al., 2004). The scale consisted of 13 items (e.g., “I am good at resisting temptations”; α = 0.63) which had to be answered on 5-point Likert-type scales, ranging from 1 (*not at all*) to 5 (*very much*). We did not expect any statistically significant differences between our experimental groups in their dispositional self-control strength.

We also measured depletion sensitivity by applying the Depletion Sensitivity Scale (Salmon et al., 2014) which contains 15 items (e.g., “I get mentally fatigued easily”; α = 0.82) getting answered on 7-point Likert-type scales (1 – *totally disagree* to 7 – *totally agree*). This scale was implemented because we wanted to analyze if the experimental groups differed in their proneness to ego deletion in the current study.

Then, self-control strength was experimentally manipulated. The self-control manipulation compromised of transcribing a neutral text for 6 min on a separate sheet of paper (Bertrams et al., 2010). In the depletion group participants were instructed to always omit the letters “e” and “n” while transcribing the text. This task requires self-control strength as individuals need to volitionally override their habitual writing tendencies when completing the task (e.g., Muraven, 2008). Participants from the non-depletion group transcribed the same text conventionally as no additional instructions were given, which does not require use of self-control strength. Therefore, in the non-depletion group, self-control strength should remain relatively intact as opposed to the depletion group (cf. Schmeichel, 2007; Bertrams et al., 2010).

To determine the validity of the self-control manipulation participants were required to answer a four-item manipulation check (e.g., Englert and Bertrams, 2014) related to the transcription task (“How difficult did you find the task?” “How effortful did you find the task?” “How mentally depleted do you feel at the moment?” “How strongly did you have to regulate your writing skills?”; α = 0.80) on Likert-type scales ranging from 1 (*not at all*) to 7 (*very much*). The transcribed texts were also analyzed for the following variables: number of characters transcribed and percentage of errors made.

Directly following the manipulation check participants were asked to complete another series of three 20 m sprints at maximal effort (T2). The procedure was identical to the baseline measurement (T1). Again, we measured reaction time and number of false starts via the Brower timing system.

Once the experiment was completed, participants were debriefed, asked if they had any questions, and thanked for their participation.

## Results

### Preliminary Analyses

The results of the following analyses are displayed in Table 1. Analyses of variance (ANOVA) revealed that the depletion and non-depletion group did neither differ in their trait self-control strength, *F*(1, 36) = 0.82, *p* = 0.37, ${\text{η}}_{p}^{2}$ = 0.02, nor in their depletion sensitivity, *F*(1, 36) = 2.58, *p* = 0.12, ${\text{η}}_{p}^{2}$ = 0.07.

**TABLE 1:** **Descriptive statistics: means and standard deviations**.

****	**Group**			
	**Depletion**		**Non-depletion**	
**Variable**	***M***	**SD**	***M***	**SD**
SCS	3.31	0.41	3.44	0.51
DSS	3.66	0.85	3.17	0.99
Manipulation check	4.86	0.97	2.97	1.28
Transcribed characters	114.21	20.69	146.47	24.20
Number of mistakes	9.58	5.10	1.79	2.46
Number of false starts T1	0.00	0.00	0.05	0.23
Number of false starts T2	1.05	0.97	0.21	0.42

n = 19 in ego depletion group, n = 19 in non-depletion group. Overall scores of a psychometric scale were obtained by averaging the responses to the scale items. SCS = Brief Self-Control Scale; DSS = Depletion Sensitivity Scale. Number of false starts T1 = Average number of false starts out of three 20-m maximal effort sprints at first time of measurement; Number of false starts T2 = Average number of false starts out of three 20-m maximal effort sprints at second time of measurement.

In line with our assumptions, participants from the depletion group had higher scores on the manipulation check indicating that they had to invest more self-control strength while transcribing the text, *F*(1, 36) = 25.95, *p* < 0.001, ${\text{η}}_{p}^{2}$ = 0.42. In the depletion group participants also transcribed statistically significantly fewer words, *F*(1, 36) = 19.51, *p* < 0.001, ${\text{η}}_{p}^{2}$ = 0.35, and committed more mistakes, *F*(1, 36) = 35.92, *p* < 0.001, ${\text{η}}_{p}^{2}$ = 0.50. These results indicate that our experimental manipulation of self-control strength was successful.

### Main Analyses

First, we conducted a 2 (group: depletion vs. non-depletion) × 2 (time of measurement: T1 vs. T2) mixed-design ANOVA to analyze whether ego depletion affected the number of false starts. For this reason, we averaged each participant’s number of false starts, separately for T1 and T2. A significant main effect of group emerged, *F*(1, 36) = 10.33, *p* = 0.001, ${\text{η}}_{p}^{2}$ = 0.22. There was also a main effect of time of measurement, *F*(1, 36) = 23.34, *p* < 0.001, ${\text{η}}_{p}^{2}$ = 0.39. Most important, we found a significant interaction between group and time of measurement, *F*(1, 36) = 12.75, *p* < 0.001, ${\text{η}}_{p}^{2}$ = 0.26. *Post hoc* pair-wise comparisons with Bonferroni corrections showed that in the depletion group the average number of false starts significantly increased from T1 (i.e., before the ego depletion manipulation) to T2 (i.e., after the ego depletion manipulation), *F*(1, 36) = 35.29, *p* < 0.001, ${\text{η}}_{p}^{2}$ = 0.50. In contrast, there was no significant difference in the average number of false starts between T1 and T2 in the non-depletion group, *F*(1, 36) = 0.79, *p* = 0.38, ${\text{η}}_{p}^{2}$ = 0.02.

Comparison of the total number of false starts per group and time of measurement confirmed the results of the ANOVA. For the depletion group the number of false starts increased significantly from 0 to 20 from T1 to T2, *χ*^2^(1, *N* = 21) = 17.19, *p* < 0.001 while this was not the case for the non-depletion group, with 1 false start at T1 and 4 at T2, *χ*^2^(1, *N* = 5) = 1.8, *p* = 0.18. (Note that in the first chi-square analysis we substituted 1 for 0 as zeros are not accepted in the analysis).

We could not reasonably analyze average reaction times as another dependent variable. This was due to the relatively high number of false starts (i.e., our variable of primary interest) that was unequally distributed over the experimental conditions. False starts represent missing values in terms of start reaction times in our data set. (Note that our timing device did not count the reaction times of false starts but only indicated that a start was invalid).

## Discussion

In sprints in track and field split seconds can determine the finish in a 100 m dash (e.g., Harland and Steele, 1997; Santana, 2000; Gough, 2006; Pilianidis et al., 2012). This highlights the importance of initiating the sprinting movement as quickly as possible after the starting signal has been given. However, starting too early leads to immediate disqualification from competition (e.g., Ditroilo and Kilding, 2004). Therefore, an athlete on the one hands needs to inhibit the impulse of starting too early and on the other hand needs to volitionally override the tendency to wait too long. Both acts, inhibiting a movement and initiating a movement, are considered self-control acts (e.g., Richeson and Shelton, 2003; Finkel et al., 2006; McEwan et al., 2013; Graham et al., 2014). According to the strength model, self-control acts are performed less efficiently after having previously exerted self-control in a preceding task (e.g., Baumeister et al., 1998). Englert and Bertrams (2014) found that participants in a state of ego depletion showed slower average reaction times in a series of sprints as compared to a control group with available self-control strength. However, the authors did not find any effect of ego depletion on the number of false starts. The sample in that study consisted of track and field athletes with experience in the 100 m dash and the athletes were well aware of the negative consequences of a false start (i.e., immediate disqualification). That is why the authors reasoned that in experienced track and field athletes the predominant response tendency while waiting in the starting block is to make sure not to initiate their movement too soon. The ability to volitionally override this response tendency depends on self-control strength, which is why athletes in a state of ego depletion displayed slower average reaction times. In that study no false starts occurred; consequently, there was no significant effect of ego depletion on the number of false starts.

In the present study, we wanted to expand the findings by Englert and Bertrams (2014) as we conducted a similar study with a sample of athletes without any experience in track and field (i.e., female soccer players). We postulated that in inexperienced athletes the predominant response tendency in a starting block may not be to avoid a false start at any cost as the potential disqualification after a false start is not as well-elaborated in inexperienced athletes as opposed to well-experienced track and field athletes. Therefore, we assumed that depleted participants without sprinting experience would display a higher number of false starts compared to participants with available self-control strength. The results were in line with our hypotheses. Compared to baseline, more false starts occurred on average under ego depletion, but this was not the case when self-control strength was intact.

Together with the results of Englert and Bertrams (2014), the present findings indicate that the level of experience with track and field sprinting is relevant in terms of how ego depletion impairs the sprint start. When experience is high, ego depletion slows the start speed down; when experience is low, ego depletion increases the likelihood of false starts. A plausible explanation would be that high and low experienced athletes differ in how they have built a habit to avoid false starts (and the related disqualification). However, ego depletion generally seems to be detrimental to the sprint start. Therefore, regardless of experience with track and field sprint, athletes are well advised to avoid ego depletion prior to the sprint start.

We would like to discuss three limitations. First and most importantly, as previously mentioned it would not have been appropriate to analyze the average reaction times because of the fact that the high number of false starts was unequally distributed over the experimental conditions. This is why we were not able to directly contrast our findings to the findings of Englert and Bertrams (2014) in which the authors found out that ego depletion did influence reaction times in experienced track and field athletes.

Second, we did not assess participants’ mood following the transcription task designed to manipulate self-control strength. One could argue that overriding a well-elaborated behavior (i.e., omitting letters while transcribing a text) could be associated with negative emotional states in contrast to participants who did not have to exert any self-control strength. However, previous research has repeatedly demonstrated that the different instructions for the transcription task did not affect mood (e.g., Englert and Bertrams, 2012).

Third, recently there have been discussions regarding the validity of the strength model of self-control, as it has been argued that impaired self-control performance after a previous self-control act may not be caused by a temporary depletion of self-control strength. For instance, Job et al. (2010) found out that an individuals’ subjective theory about willpower has an influence on the ego depletion effect: In their study, the typical ego depletion effect was only found in participants who believed in a limited capacity of self-control strength (Job et al., 2010). In contrast, individuals who viewed self-control strength as an unlimited resource did not suffer from ego depletion. Other researchers proposed that the negative effects of a primary self-control act on subsequent self-control performance might be caused by motivational shifts (e.g., Inzlicht and Schmeichel, 2012; Inzlicht et al., 2014) or resource allocation (Beedie and Lane, 2012). Although most alternative theoretical models do not deny that self-control performance gets worse after having performed a primary self-control task, they highlight the importance of identifying the actual processes of how ego depletion impairs performance.

To conclude, our results expand previous findings by Englert and Bertrams (2014) as we demonstrated that ego depletion also has a detrimental influence on the sprint start in athletes without track and field experience. Working on a primary self-control task (in this case, a cognitive task) had a carry-over effect on a secondary self-control task from a completely different domain, in this case a gross motor task.

## Author Contributions

CE, BP, RO, and AB substantially contributed to study design. CE, BP, RO, and AB contributed to the writing of the manuscript. All authors approve the final version of the manuscript. The authors agree to be accountable for all aspects of the work in ensuring that questions related to the accuracy or integrity of any part of the work are appropriately investigated and resolved.

### Conflict of Interest Statement

The authors declare that the research was conducted in the absence of any commercial or financial relationships that could be construed as a potential conflict of interest.
